# Implementing integrated care guidelines in asthma and COPD: It ain't easy!

**DOI:** 10.1016/j.heliyon.2023.e21540

**Published:** 2023-10-31

**Authors:** Jan A. Witte, Erwin Birnie, Gert-Jan Braunstahl, Edmée van den Akker, Walter J.M. van Litsenburg, Niels H. Chavannes, Maureen P.M.H. Rutten - van Mölken, Johannes C.C.M. In ’t Veen

**Affiliations:** a Department of Pulmonary Disease, Franciscus Gasthuis & Vlietland, Rotterdam, the Netherlands; b Department of Pulmonary Disease, Erasmus MC, Rotterdam, the Netherlands; c Department of Pulmonary Disease, Catharina Ziekenhuis, Eindhoven, the Netherlands; d Department of Public Health and Primary Care, Leiden University Medical Center, Leiden, the Netherlands; e Institute of Health Care Policy and Management/Institute of Medical Technology Assessment, Erasmus University Rotterdam, Rotterdam, the Netherlands

## Abstract

**Objective:**

To evaluate the implementation of a guideline-based, integrated, standardised, personal approach in patients with Chronic Obstructive Pulmonary Disease (COPD) or Asthma in a real-life situation.

**Methods:**

Patients at the outpatient clinic of the department of pulmonary disease were included in a controlled cohort study, comparing the use of diagnostic items and ‘Personalised care plans' (PCPs) in patients with obstructive lung disease before (2013) and after (2015) implementation of a personalised diagnostic pathway. Results were compared with reference data (2016) from two control hospitals that used the same guidelines but did not implement this pathway.

**Results:**

100 patients were selected for all three cohorts. After implementing the diagnostic pathway in 2015, 35 % of patients visited attended all pre-planned appointments, whereas 65 % of patients did not: they were diagnosed using usual care. Factors contributing to patients not attending the diagnostic care pathway were: the logistical complexity and intensity of the 2-day pathway, patients willingness to participate in a personalised pathway, and low social economic status or low literacy. After the implementation of the pathway, a significant improvement was seen in the number of PCPs (P < 0.001) and the number of diagnostic items registered recorded in the patients' electronic medical records (P < 0.001).

**Conclusion:**

Implementing a standardised diagnostic pathway in a real-life population significantly improved the number of personalised care plans, demonstrating that the implementation of holistic care planning is feasible in this population. Nevertheless, the pathway needs further improvements to maximize the number of patients benefitting from it, including logistical streamlining, removing unnecessary diagnostic tools, and increasing the focus on low literacy. Additionally, we found that implementing existing guidelines in a real life context is complex. Therefore, it is required to prioritize the translation of current guidelines into every-day practice, before expanding existing guidelines and protocols.

## Highlights

• Implementing a holistic and structured diagnostic pathway in patients with asthma or COPD is feasible.
• This diagnostic approach should consist of three items:
  • Assessment of diagnosis and health status focused on treatable traits
  • Structured summarization of outcomes in the electronic medical records.
  • Development of a holistic approach within a multidisciplinary setting leading to a personalised care plan.
• The introduction of new pathways enhances the quality of care for patients engaged in the pathway as well as those receiving standard care.
• New care pathways could benefit from a standardised yet personalised and adaptable approach.
• Motivational status, willingness to change, and health literacy are pivotal parameters for future diagnostic pathways.
• New guidelines must include a chapter describing its implementation.

## 1 Introduction

Asthma and Chronic Obstructive Pulmonary Disease (COPD) are highly prevalent chronic obstructive lung disorders, which often impact multiple facets of affected people's lives, including their emotional well-being, social interactions, and overall quality of life. Traditional diagnostic pathways focussed on physiological measurement, such as lung function and blood tests, however, physiological outcomes cannot fully explain a patients well-being [1,2]. Therefore, holistic care models are necessary to capture both physiological measurement and other components affecting the quality of life, such as functional limitation, comorbidities and other symptoms. The chronic care model (CCM) is such a model that aims to improve prevention, diagnosis, and management in patients with a chronic diseas. Various chronic care models have been proposed, identifying at least 5 essential components: per-patient tailered care, patient self-management abilities, multidisciplinary approach, patient oriented logistical infrastructure and implementation of electronic devices [3]. However, although these essential components are often included in international guidelines and protocols, they often lack a practical description of its implementation in a real life context [[4], [5], [6], [7], [8], [9], [10], [11]]. Our group therefore performed a Delphi panel, asking 153 pulmonologists, respiratory nurses, general practitioners, advisors of health insurance companies, and delegates of patient organizations to establish a collective opinion on 41 diagnostic tests and other compoments in a 2-round Delphi study [12]. The panel agreed on 29 out of 41 components and grouped these in 4 main domains: physiological functioning, symptoms, functional limitations, quality of life. These items were subsequently integrated in a dianostic care pathway, aiming to achieve a personalised care plan for all newly reffered patients with COPD or asthma [13]. Here we evaluate the implementation of a guideline-based, integrated, standardised, yet personal approach which uses documented treatable traits to improve the number of Personalised Care Plans (PCPs) in patients with COPD or Asthma in a real-life situation.

## 2 Methods

### 2.1 Outline of the diagnostic care pathway

The diagnostic pathway was implemented for newly referred patients with asthma and COPD. The details of the implementation strategy have been published before [13]. In short, referred patients underwent an initial screening to determine if the general practitioner suspected an obstructive pulmonary condition. Subsequently, patients visited both a Pulmonologists and a Nurse Physician to discuss symptoms and diagnosis, prescribe medication, optimize inhaler technique and start measurements with a triaxial accelerometer. Patients revisited after 1 week to perform lung functions tests and return the triaxial accelerometer. Finally, patients visited the hospital again after 6 weeks to interpret measurements and create a PCP. A flowchart of this pathway has been published before.

### 2.2 Diagnostic tools of the diagnostic pathway

The assessment was carried out during 2 visits, 7 days apart, as the move monitor measured movements for multiple days. It consisted of an evidence-based set of measurements prioritised by a Delphi procedure we conducted earlier [4,5,12]. This set of measurements includes traditional items such as prior medical history, physical examination, spirometry, blood gases and X-ray. Additionally, four innovative diagnostics were implemented after a Delphi panel study.

1. The metronome-paced hyperventilation test measures dynamic hyperinflation, as dynamic hyperinflation contributes to the feeling of dyspnoea but often goes unrecognised [14];
2. A 3D axial activity monitor objectively evaluates the physical activity in daily life, as impaired physical activity is a prognostic marker for asthma control, disease-specific quality of life and mortality; The activity monitor registers movement for approximately one week [[15], [16], [17]].
3. The Nijmegen Clinical Screening Instrument is a comprehensive assessment tool to acquire detailed insight into symptoms, functional limitations, and quality of life [7,13].
4. A multidisciplinary consultation between Chest Physicians and Nurse Practitioners to discuss the attained diagnostic information and subsequently identify treatable traits, according to the four-domain model of physiological impairment, functional impairment, symptoms and quality of life.

### 2.3 Implementation strategy

The multidisciplinary team involved in the implementation consisted of pulmonologist, physician assistant, specialist nurse, physical therapist, lung function technician, members of the respiratory management unit, and a member of the administrative staff of the pulmonology department. Each discipline (separately) received training on relevant areas. For example, pulomonologists were trained on screening of newly referred patients and interpretation of data, Nurse physicians were trained on the creation of PCPs and the management unit was trained on the logistics of the pathway. Representatives from each discipline convened regularly to discuss the progress and barriers of the care pathway and make changes where needed.

### 2.4 Study subjects

Incluison criteria.

• Patients referred to secondary care
• Suspected diagnosis of obstructive lung disease
• Aged 40 or older

Exclusion criteria.

• Severe interfering mental or physical comorbidity
• Unable to read or understand Dutch

### 2.5 Data collection

Data was colleted from 3 arms.

1) A retrospective cohort to determine the baseline situation (Franciscus Hospital, Rotterdam, 2013)
2) A prospective cohort after implementation of the new pathway (Franciscus Hospital, Rotterdam, 2015) [13,[18], [19], [20]].
3) A retrospective cohort to determine national trends regarding a holistic diagnostic approach (2016, 2 large teaching hospitals in the Netherlands)

In a controlled but not randomised cohort study, we compared the implementation of our evidence-based diagnostic pathway in patients with asthma or COPD before (2013) and after (2015) the implementation period of the diagnostic pathway at the pulmonology outpatient clinic. Implementation was measured by the change in the number of diagnostic items addressed in the patients' health records and the change in the number of Personalised Care Plans (PCPs). A PCP is a dynamic action plan created by the patient and healthcare providers, focussing on patient goals, needs, motivation, and shared decision-making. It represents both the process of collaborative goal-setting and the structured documentation addressing factors like risk mitigation, medication usage, and the monitoring of health metrics.

All patients referred for diagnosis and treatment of asthma or COPD were eligible for inclusion to avoid selection bias. For the 2013 cohort, 100 medical records of patients referred to the outpatient clinic were selected by an independent researcher using a random number generator. For the 2015 cohort, all newly referred patients were invited for the 2-day diagnostic pathway, of which the first 100 patients were randomly selected for this study. Retrospective data from two large control hospitals were used to determine national trends regarding implementation.

To avoid bias due to updated guidelines and general improvements in health care between 2013 and 2015, we selected two outpatient clinics in the Netherlands to provide a control group. A test-retest study was done to ensure similar database development between these hospitals and our hospital. The inter-observer variability was very low, with a substantial Cohens kappa of 0.97 [21].

Variables derived from the Delphi rounds were in 4 diagnostic domains: 18 items for physiological functioning, 4 items for symptoms, 3 items for functional limitations, and 2 items for quality of life. Data was collected in a binary manner for all of the diagnostic items (present or not present in electronic medical record), apart from age and diagnosis which were numerical and categorical variables respectively.

### 2.6 Primary endpoint

This studies primary endpoint was the difference in PCPs created before and after introduction of the diagnostic pathway.

### 2.7 Statistics

Cohort characteristics were created for all cohorts using descriptive statistics. Descriptive data for the primary outcome were described using medians and Inter Quartile Range (IQR).

The number of diagnostic items assessed per patient was calculated and compared between the baseline cohort (2013), diagnostic care pathway cohort (2015), and control hospital cohort (2016) using the Mann-Whitney *U* Test. The proportion of PCPs between these cohorts will be compared with the independent samples proportion test.

## 3 Results

**Fig. 1:**
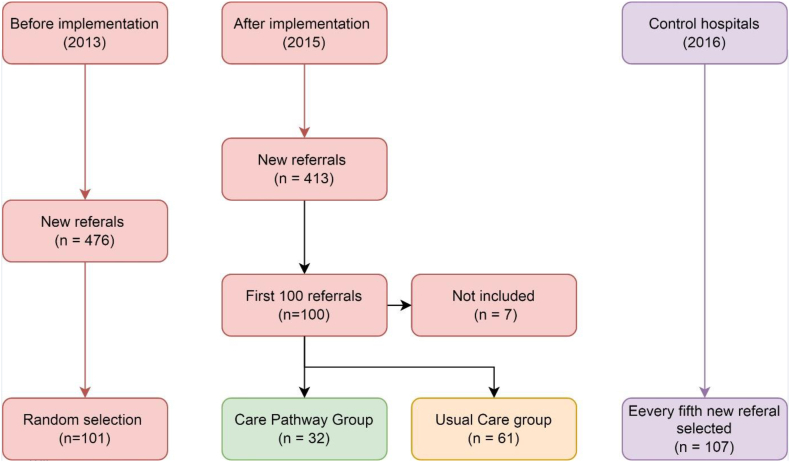
The intervention hospital included new referrals during two periods; 2013 and 2015. Additionally, in 2016 two control hospitals participated by including every fifth new referral. The intervention hospital implemented the 2-day assessment in 2015, which resulted in two groups; one group completed the 2-day assessment and one group did not complete the 2-day assessment. Finally, four groups were analysed.

Upon referral, 100 patients were allocated for treatment in the 2-day diagnostic pathway of 2015 (Fig. 1). One physician couldn't assess 7 patients due to retirement: those patients were excluded from our analysis. A total of 32 patients (35 %) were diagnosed using the 2-day diagnostic pathway, and the remaining 61 patients (65 %) could not be diagnosed by this structured pathway. These 61 patients were diagnosed conventionally based on the attending pulmonologists' individual opinions and the availability of timeslots for appointments. The baseline characteristics can be found in Table 1.

**Table 1:** Patient characteristics before and after implementation of the 2-day diagnostic pathway.

Patient features		Before implementation (2013)	After implementation (2015)		
			All patients	Diagnostic pathway	Usual care
N =		101	93	32	61
Mean age, years (SD)		53,82 (±16,08)	57,04 (±17,17)	56,63 (±16,24)	57,26 (±17,76)
sex, n (%)	Male	43 (43 %)	38 (41 %)	10 (31 %)	28 (46 %)
	Female	58 (57 %)	55 (59 %)	22 (69 %)	33 (54 %)
Diagnosis, n (%)	COPD	16 (16 %)	32 (34 %)	8 (25 %)	24 (39 %)
	Asthma	45 (45 %)	45 (48 %)	15 (47 %)	20 (49 %)
	ACO	4 (4 %)	6 (6,5 %)	5 (16 %)	1 (2 %)
	Other	36 (36 %)	9 (10 %)	3 (9,4 %)	6 (10 %)

The creation of PCPs was significantly increased in the 2015 cohort compared to the baseline (2013) and control (2016) cohorts (Table 2, Table 3). Additionally, registration of the diagnostic tools within the 4 domains increased significantly (P < 0.001, Table 4).

**Table 2:** Number of PCPs created.

Domain	Baseline	Control hospitals	Usual Care	Care Pathway
Personalised Care Plan, n(%)	2 (2 %)	0 (0 %)	27 (44 %)	17 (53 %)

**Table 3:** Median percentage (IQR) of items from the 4-domain model addressed in electronic health records of patients with asthma or COPD.

Domain	Baseline	Control hospitals	Usual Care	Care Pathway
Physiological impairments	61 % (17 %)	67 % (11 %)	72 % (11 %)	78 % (15 %)
Symptoms	75 % (25 %)	50 % (50 %)	75 % (25 %)	75 % (25 %)
Quality of life	0 % (0 %)	0 % (0 %)	100 % (0 %)	100 % (50 %)
Functional limitations	0 % (0 %)	0 % (33 %)	67 % (33 %)	33 % (67 %)
Overall	54 % (19 %)	54 % (8 %)	69 % (12 %)	74 % (16 %)

**Table 4:** Statistical differences between groups, as compared to the Care Pathway group.

		Baseline (2013)	Usual Care (2015)	Control Hospitals (2016)
Domains	Physiological impairments	<0,01	<0,01	<0,01
	Symptoms	<0,01	0,517	<0,01
	Quality of life	<0,01	0,06	<0,01
	Functional limitations	<0,01	0,648	<0,01
	Overall domains	<0,01	0,092	<0,01
Personalised Care Plan		<0,01	0,416	<0,01

**Fig. 2:**
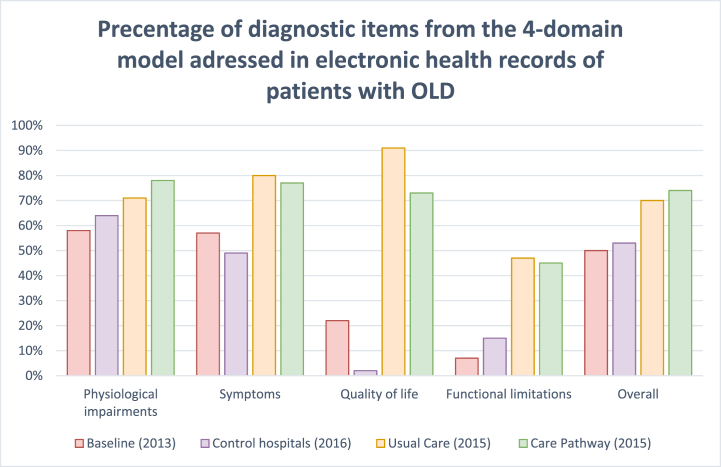
The number of assessed components per domain per group.

The 2015 cohort consisted of 32 patients assessed in the 2-day pathway (care pathway group) and 61 patients not (usual care group). The number of PCPs created did not differ statistically between these groups (53 % vs. 44 %; p = 0.416). Furthermore, attention for the 4 diagnostic domain domains was similar between the care pathway group and the usual care group (74 % vs 69 %; p = 0.092), except for a significant difference in the physiological impairments domain (78 % vs. 72 %; P = 0.001) and a trend to significance in the Quality of Life domain (P = 0.06), see Fig. 2.

In the 2015 cohort, approximately 10 % of referred patients could not be diagnosed with either asthma and/or COPD.

## 4 Discussion

In this study, we evaluated the implementation of a standardised diagnostic pathway for patients suspected of Asthma and/or COPD. We found that the implementation of a holistic diagnostic pathway is feasible in this population as the number of PCPs increased significantly. However, we found that implementing existing guidelines in a real life context is complex, which led to a significant group of patients not completing the pathway.

A total of 100 patients were allocated to the 2-day diagnostic pathway, evenly distributed among 5 physicians. One of the physicians could not assess 7 out of 20 patients. Therefore, only 93 patients were analysed. A significant number (n = 61; 65 %) of patients could not attend all study visits. The reasons were not recorded structurally, however, problems were reported in two domains: the medical team and the patients perspectives. Firstly, the medical team reported that the sequence of appointments was too rigid for the staff at the outpatient clinic. As a results, one or more appointments could not be planned within the allocated time frame. Additionally, staff member suspected low healthy literacy in some patients, resulting in patients not filling in questionnaires and missing planned appointment. Secondly, patients reported that the 2-day assessment was too intensive, had too many appointments and some meauresement were too intimate (for example questions on sexual health or the 7-day physical activity tracker). A prior study had comparable results, as 62 % of patients did not return for a follow-up visit [22]. Most of these patients did not return for unknown reasons (39 %). The remaining no-shows were caused by logistics (22 %) and death (<1 %). This demonstrates that implementation can be challenging in patients with severe chronic obstructive lung disease. A recent study on Parkinson's disease also described this problem by showing that less than 14 % of participants completed their integrated care program [23]. The factors contributing to their disappointing numbers were related to medical staff engagement, logistics, and patient commitment.

The first step in this pathway involved the selection of new referrals for the diagnosis and treatment of obstructive lung disease. We discovered that 30 % of patients in the cohort did not have Asthma or COPD. The disparity between primary care and secondary care diagnoses has been previously described. For example, a Swiss cohort comprising 11,423 primary care patients found that reversibility was present in one-fifth of the current asthma population and in one-tenth of the ever asthma population [24]. A prospective trial conducted by our research group yielded similar results, with 41 % of newly referred patients suffering from Class III obesity and asthma was not reversible during spirometry (i.e., overdiagnosis). Additionally, 31 % of patients with obesity but without a prior asthma diagnosis were newly diagnosed with asthma (i.e., underdiagnosis). The difference in diagnostic accuracy between primary care and secondary could be attributed to multiple factors. For example, secondary care asthma is often diagnosed in a lung function laboratory with specialized equipment and specialized lung function technicians, whereas primary care asthma often relies on hand-held devices and general nurse practitioners. Furthermore, primary care predominantly uses spirometry, whereas secondary uses additionaly diagnostic tools such as bronchial challenge tests and impuls oscillometry to diagnose asthma. Bronchial challenge test (BCT) could be useful in primary care to increase diagnostic sensitivity and potentially reduce the number of incorrect refferals to secondary care. The feasibility of BCT has been studied before by Bins et al. using 5 years of retrospective data from a Medical Diagnostic Center in Rotterdam, which is the only primary care diagnostic center to perform direct BCT with histamine in Netherlands [25]. Their findings indicated that roughly half of the referred patients had a high pre-test probability of asthma, of which 75 % tested positively in the BCT, thus achieving an accurate asthma diagnosis. The correct diagnosis of asthma in these patients could potentially obviate the need for a secondary care referral, thereby reducing the costs incurred in secondary care.

Before the implementation of this pathway, diagnostic strategies varied among physicians. As a result, diagnostic outcomes were not recorded structurally in an electronic medical record and care plans were not personalised. After implementing the 2-day pathway, diagnostic outcomes were recorded structurally and adequately in the patients' electronic medical record, resulting in significantly more patients provided with a Personalised Care Plan. Furthermore, the focus on physiological impairments and quality of life increased significantly, emphasising the value of structured diagnostic pathways for Personalised Health Care.

In this study, we did not intend to create new guidelines: instead, we aimed to implement existing standards in day-to-day practice using a new holistic diagnostic approach. This implementation led to a significant increase in the percentage of patients with a Personalised Care Plan. Interestingly, these improvements were observed even among patients unable to attend all visits in the 2-day assessment. This was discussed during periodic multidisciplinary meetings with the project team, after which a more flexible approach was used in these patients, basically removing the strict 2-day schedule without removing any measurements and visits. As a result, the number of PCPs increased in this group. This highlights the significance of recurrent meetings and education, which equipped the project team to navigate such adjustments.

Conversely, we found it challenging to overcome logistical complexities and gain patients' commitment to a new approach. Logistical problems arising from the tight 2-day schedule must be addressed in future iterations of the pathway by using a more flexible approach, both in terms of patients preferences as well as logistical preferences from the medical staff. Moreover, the suspicion of health illiteracy emerged on multiple occasions. after discussing this issue in a perodic multidisciplinary meeting, we planned to integrate a graphical questionnaire for patients with possible healthcare illiteracy. Following discussions in periodic multidisciplinary meetings, we devised plans to incorporate a graphical questionnaire for patients with potential healthcare illiteracy. Our journey began with the creation of a graphical CCQ (CCQg), the results of which are soon to be published and will be presented at the upcoming ERS congress.

Furthermore, patients commitment could be assessed by implementing a willingness to change questionnaire during enrolment. This approach would focus on motivation, opportunity, and capacity of patients, according to the Poiesz triad [26]. Additionaly, several implementation guidelines have been published recently, which could benefit future guidelines [[27], [28], [29]].

Several strengths of this study have to bo mentioned. First of all, we created and published an implementation strategy that began with all relevant stakeholders. These stakeholders gave imput on the different aspects of a diagnostic pathway in a 2-round Delphi round. This Delphi round was strengthend by the presence of both primary and secondary care phycisians. We therewith created a secondary care diagnostic pathway that was ultimatiliy beneficial for the reffering general physician. Furthermore, we included two dutch teaching hospital in addition to the baseline cohort. This way, we could correct for possible national trends regarding COPD and asthma care. However, this comparison had its limitations, as the two control hospitals included significantly more patients with COPD in the project. As a result, the mean age was higher in these hospitals. This reduced the comparibility between these hospitals. Additionally, we did not structurally report the reason of drop-out from the 2-day program, which we will implement in future iterations of this pathway.

## 5 Conclusion

Implementing existing guidelines in newly referred patients with asthma or COPD in a standardised diagnostic pathway is feasible in a real-life population. However, several barriers need to be addressed in future iterations of the pathway, such as logistigical streamlining, removal of unnecessary diagnostic tools and development of tools for patients with low health literacy. Furthermore, we should focus on implementing existing guidelines rather than creating new ones, as implementation is one of the most challenging aspects of new pathways and protocols.

## Funding information

Picasso voor COPD, Astra Zeneca.

### Ethics approval and consent to participate

This study was performed after approval by the Institutional Research Board of the Franciscus Gasthuis en Vlietland, Rotterdam, the Netherlands with number RvB/KK/cy/2018–108/T110. This research was declared as outside the scope of the Medical Research Involving Human Subjects Act, because routinely collected health care data were used after pseudonymization. This waives the requirement to obtain informed consent.

### Availability of data and material

The data supporting the findings of this study are available from Franciscus Gasthuis en Vlietland but restrictions apply to the availability of these data, which were used under license for the current study, and so are not publicly available. Data are however available from the authors upon reasonable request and with permission of all authors.

## CRediT authorship contribution statement

**Jan A. Witte:** Writing – review & editing, Writing – original draft, Visualization, Software, Formal analysis. **Erwin Birnie:** Writing – review & editing, Formal analysis, Data curation, Conceptualization. **Gert-Jan Braunstahl:** Writing – review & editing, Supervision, Methodology, Investigation, Data curation, Conceptualization. **Edmée van den Akker:** Writing – original draft, Methodology, Investigation, Formal analysis, Data curation, Conceptualization. **Walter J.M. van Litsenburg:** Writing – review & editing, Writing – original draft, Resources, Project administration, Methodology, Formal analysis, Data curation. **Niels H. Chavannes:** Writing – review & editing, Writing – original draft, Resources, Project administration, Methodology, Investigation, Funding acquisition, Data curation, Conceptualization. **Maureen P.M.H. Rutten - van Mölken:** Writing – review & editing, Writing – original draft, Methodology, Formal analysis, Conceptualization. **Johannes C.C.M. In ’t Veen:** Writing – review & editing, Writing – original draft, Supervision, Resources, Project administration, Methodology, Investigation, Funding acquisition, Formal analysis, Conceptualization.

## Declaration of competing interest

The authors declare that they have no known competing financial interests or personal relationships that could have appeared to influence the work reported in this paper.
